# Proactive vs. reactive country responses to the COVID-19 pandemic shock

**DOI:** 10.1371/journal.pgph.0001345

**Published:** 2023-01-24

**Authors:** Pier Luigi Sacco, Francesco Valle, Manlio De Domenico

**Affiliations:** 1 DiSFiPEQ, University of Chieti-Pescara, Pescara, Italy; 2 metaLAB (at) Harvard, Cambridge, Massachusetts, United States of America; 3 Fondazione Bruno Kessler, Povo, Italy; 4 Department of Physics and Astronomy “Galileo Galilei”, University of Padova, Padova, Italy; 5 Padua Center for Network Medicine, Padova, Italy; Management Sciences for Health, UGANDA

## Abstract

The infection caused by SARS-CoV-2, responsible for the COVID-19 pandemic, is characterized by an infectious period with either asymptomatic or pre-symptomatic phases, leading to a rapid surge of mild and severe cases putting national health systems under serious stress. To avoid their collapse, and in the absence of pharmacological treatments, during the early pandemic phase countries worldwide were forced to adopt strategies, from elimination to mitigation, based on non-pharmacological interventions which, in turn, overloaded social, educational and economic systems. To date, the heterogeneity and incompleteness of data sources does not allow to quantify the multifaceted impact of the pandemic at country level and, consequently, to compare the effectiveness of country responses. Here, we tackle this challenge from a complex systems perspective, proposing a model to evaluate the impact of systemic failures in response to the pandemic shock. We use health, behavioral and economic indicators for 44 countries to build a shock index quantifying responses in terms of robustness and resilience, highlighting the crucial advantage of proactive policy and decision making styles over reactive ones, which can be game-changing during the emerging of a new variant of concern.

## Introduction

The organization of human societies is becoming increasingly complex and multilayered [1–3]. As a consequence, we are experimenting unprecedented levels of global interdependence through feedback loops at all scales and across all spheres of human activity [4, 5]. In this context, evidence-based evaluation approaches need to assess the impact of policies in multiple, inter-related domains, building upon suitable structural models. This is a very challenging goal, that cannot be pursued within a limited disciplinary realm, but calls for real trans-disciplinary collaboration. Whatever their area of reference, policies may have complex economic, social, environmental, and even mental health consequences, among others [6, 7]. There is a need to build evaluation frameworks that span such inter-dependencies in their relationship with policy goals. One of the most serious acid tests in this regard is provided by policy response to large-scale events with sudden, dramatic socio-economic and health consequences. Global pandemics are an obvious case in point, as they impact practically every aspect of human livelihood, often with profound transformational effects [8, 9]. Due to the COVID-19 crisis, we are currently witnessing one such a watershed moment in history, and this provides us with unique opportunities to develop and test a complexity-informed approach to policy evaluation, to support policymakers facing tough decisions that need prompt, effective responses. The present paper offers one of the first contributions toward the development of a methodology for multi-sector evaluation of the response strategies to a pandemic shock and of the related policy trade-offs.

As it is well known, in January 2020, China reported a fast increase in the number of cases of a new, severe and acute respiratory syndrome due to a novel coronavirus, the SARS-CoV-2 [10]. In less than 3 months, the pathogen spread at a global scale, leading to the COVID-19 pandemic [11–15]. The new disease was characterized by both asymptomatic and pre-symptomatic phases, leading to systematic under-detection of cases that threatened epidemic control [16]. The virus—exhibiting similarities with SARS and MERS pathogens [17], causing systemic disorders [18] through pan-viral disease mechanisms [19]—was responsible for a communicable disease characterized by a basic reproduction number of 2–3.5 [20], high enough for a sustained community transmission that could potentially overwhelm even the most prepared public health systems. In the absence of suitable medical treatments and vaccines, local governments opted for the introduction of a variety of non-pharmacological interventions (NPI), ranging from physical distancing to mandatory mask wearing, and in many cases to draconian measures such as travel bans and national lockdowns [21]. The impact of containment strategies was readily analyzed [20, 22–25] to assess the relative effectiveness of available interventions [26] and identify clear change points in the time course of epidemiological signals [27].

On the one hand, some countries adopted a proactive response, devising clear anticipatory steps to take in order to achieve a transient elimination strategy, limited to localization in their geographic areas. Proactive responses might include a spectrum of actions, from early suppression [28] to the introduction of non-pharmaceutical [29] and pharmaceutical interventions, such as indirect protection of children from infection through parental vaccination [30]. On the other hand, many other countries adopted a reactive response, devising procedures to avoid the overwhelming of their national health systems while allowing the virus to spread. In the following, we will concisely refer to these two approaches as proactive vs. reactive—or, equivalently, elimination vs. mitigation—strategies, respectively, although it is worth remarking that by “elimination” here we refer to a transient effect, subject to future reintroduction due to cases imported from other countries where the disease remains above the surveillance baseline. Therefore, we remark that neither positive nor negative interpretations of these terms are intended since, not surprisingly, both approaches had a substantial impact on human activities and national socio-economic systems, and are still largely debated [31, 32]. We refer the interested reader to Ref. [33] for further details about the principles of disease elimination and eradication.

At the social level, the non-pharmacological measures have severely restricted social interaction with negative consequences on mental health [34], and with huge personal and collective costs [35]. At the economic level, the pandemic has caused a collapse of production and business activity, pushing many companies of any size, from very large to very small, on the brink of bankruptcy [36], and exacerbating socio-economic inequalities [37]. Such negative effects are likely to persist at least partly in the long term [38]. At the educational level, the crisis has disrupted educational programs, with especially negative impacts on students with socio-economically fragile backgrounds [39].

However, in terms of evaluation frameworks, the abundant literature that has been produced to assess the impacts of the pandemic has generally focused on specific aspects. Many studies have put the socio-economic consequences under the spotlight [40–42]. Other studies have consider other major aspects such as air passenger traffic [43], tourism [44], and work organization models [45], among others. Some studies also considered key policy trade-offs such as that between socio-economic losses and environmental gains [46], economic and health outcomes [47], or safety versus poverty relief [48]. However, no comprehensive evaluation framework that provides a comprehensive assessment of the multi-sector consequences of pandemic response strategies has been provided so far to our knowledge. In this paper, we introduce a new index that allows such kind of assessment while enabling international comparisons in terms of overall response effectiveness. Such comparisons are of great importance in providing feedback to policymakers about the relative effectiveness of alternative policy options and in improving the policy response learning curve for complex, large-scale events.

## Materials and methods

### Existing pandemic indicators

Our measures of robustness and resilience are computed from a shock index built on government, health, economic and behavioral indicators combined together by iterative convolutions, detailed in the following.

#### Closure and health indices

Our government indices are based on different indicators collected by the Oxford COVID-19 Government Response Tracker team [49] and include information about restriction and containment policies as well as testing, tracking and vaccination policies. We use those indicators to create two non-overlapping indices that score between 0 and 100, exploiting the procedure described by the Oxford team, and we then apply a 7-days rolling mean on them.

#### Economic index

The economic index we use is the OECD Weekly Tracker of GDP growth [50] that proxies the percent change in weekly GDP levels from the pre-crisis trend. We scaled this index between 0 and 100, where 100 is the maximum negative percent change across all countries in a given period.

#### Behavioral index

The behavioral index is based on Google mobility data [51] and is computed by the median of the percent change from baseline value of retail and recreation, grocery and pharmacy, parks, public transport stations, workplaces and residential displacements. We then applied a 7-days rolling mean within each country and the resulting value is scaled between 0 and 100, where 100 is the maximum negative value across all countries in a given period.

#### Epidemic index

The epidemic index for a country is equal to the cumulative number of confirmed Covid-19 deaths [49] divided by the total population in 2020 [52] multiplied by one million. Such value is then scaled between 0 and 100, where 100 is the maximum across all countries in a given period. The value of the index is retained weekly to match the granularity of the other data sources.

Notice that our choice allows us to effectively compare the impact of the pandemic across countries in terms of confirmed fatalities due to COVID-19. It might be desirable also to perform a comparison with respect to a country’s baseline: in this case one could consider other indicators such as estimates of excess mortality across time. Here, we decided to exclude excess mortality from the set of indicators because its estimation is very sensitive to the choice of a reference period and, to the best of our knowledge, there is still no general consensus about which period and estimation procedure should be used to this purpose.

### Comprehensive shock index

We started by convolving two of the above indices (Closure and Economic) and then using the result as input for a second convolution with another index in an iterative fashion. Then we selected the date corresponding to the maximum value of the final convolution as a splitting point of the area under the convolution: robustness is the area under the convolution from the starting point to the “peak”, while resilience is the area under the convolution between the “peak” and the ending point of the time window considered. Both areas are estimated by numerical integration. Standardized values of robustness and resilience were then computed by subtracting from one the ratio between the value of each measure and its maximum.

Note that, here, the convolution of two functions is defined as the integral of their product after one is reversed and shifted. We started by convolving the Closure and Economic Index functions as follows

$$\begin{array}{c} \left(c*e\right)\left(t\right)=\int_{-\infty }^{\infty }c\left(\tau \right)e\left(t-\tau \right)d\tau \end{array}$$ (1)

where *c* and *e* are the Closure and Economic Index functions, respectively, and *t* and *τ* represent time. Then we applied the same formula using the result function of the previous step and the Health Index function as the two functions to convolve, and so on, until we obtain a single shock measurement.

Concerning the indicators dynamics, we divided our observation period in four overlapping time windows with increasing length of 19 weeks starting from February 23, 2020. For each time window we re-computed the scaled indices and the corresponding final convolution and peak date to get our robustness and resilience metrics, that were finally standardized as mentioned above.

More specifically, time windows have been chosen to reflect the different stages of the COVID-19 pandemic, while maintaining its global evolution and dynamics. Thus, we started by splitting the observed period into 4 equal parts of 19 weeks (2 of high emergency and 2 of low emergency). The first 19 weeks cover the first epidemic wave. The second window extends the first one by another 19 weeks, so that in the same time window we could observe both higher and lower levels of emergency, and so forth, so that the length of the fourth time window is almost one year and a half.

### Mathematical model of the evolution of a shock

**Fig 1:**
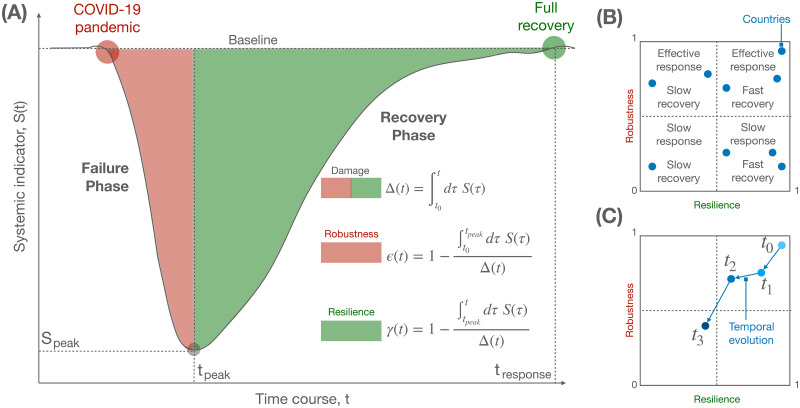
Modeling the response of a complex system to a shock. (A) Schematic of changes in a complex system—e.g., the economy of a country—as quantified by a systemic indicator—e.g., the change in GDP growth (see the text for details)—undergoing a decrease, corresponding to a failure phase, and an increase, corresponding to a recovery phase, after a shock like COVID-19. The shaded areas under the curve allow to define a measure of robustness and resilience, which can be used to quantify the response of a country. (B) The two indices are scattered to define 4 distinct types of response within a fixed temporal window, combining effective or weak robustness with fast or slow resilience. (C) Similar to (B), but considering the temporal evolution of the two indices for a given country, allowing to monitor the trend of the system over time.

As outlined in Fig 1, we characterize the evolution of a shock by means of two distinct phases:

1. *Failure phase*: where the shock induces perturbations decreasing the system’s function or, equivalently, increasing the system’s dysfunction through an expansion.
2. *Recovery phase*: where interventions to mitigate the failures induces new perturbations increasing the system’s function or, equivalently, decreasing the system’s dysfunction through a contraction.

Given the nature of complex adaptive systems, it is plausible to assume that growing and shrinking dynamics are governed by some kind of multiplicative process, which is the only fundamental assumption of our model.

During the expansion phase—where failures are detrimental for the system’s function—changes in the dysfunction indicator $D_{fail}\left(t\right)$ are assumed to be proportional to the value of a suitably normalized measure indicator *y*(*t*), which in turn it is subject to a logistic shrink:

$$\begin{array}{c} \left\{ \begin{array}{c} {\dot{D}}_{fail}\left(t\right)=By\left(t\right)\\ \dot{y}\left(t\right)=By\left(t\right)\left[y\left(t\right)-1\right] \end{array}\;\right. \end{array}$$ (2)

leading, for the boundary condition $y\left(0\right)=\frac{1}{2}$, to

$$\begin{array}{c} {\dot{D}}_{fail}\left(t\right)=\frac{B}{1+e^{Bt}}. \end{array}$$ (3)

The choice of the logistic dynamics is motivated by thinking about failures in terms of a population growth which is sustained, with rate *B*, by available resources until a carrying capacity *K* is reached. To keep the model as simple as possible, we consider that *K* = 1, since maximum failures are represented by the normalized measure *y*_*max*_ = 1.

Conversely, in the contraction phase, where recovery takes place, changes in the dysfunction indicator $D_{reco}\left(t\right)$ are assumed to be proportional to the value of a suitably normalized measure indicator *x*(*t*), which in turn it is subject to a logistic growth:

$$\begin{array}{c} \left\{ \begin{array}{c} {\dot{D}}_{reco}\left(t\right)=-Ax\left(t\right)\\ \dot{x}\left(t\right)=Ax\left(t\right)\left[1-x\left(t\right)\right] \end{array}\;\right. \end{array}$$ (4)

leading, for the boundary condition $x\left(0\right)=\frac{1}{2}$, to

$$\begin{array}{c} {\dot{D}}_{reco}\left(t\right)=-\frac{A}{1+e^{-At}}. \end{array}$$ (5)

As for the failure phase, the choice of the logistic dynamics is motivated by thinking about failures in terms of a population shrink which is sustained, with rate *A*. Also in this case *K* = 1, since *x*_*max*_ = 1.

Since the two dynamics of failure and recovery compete with each other, the overall dysfunction indicator can be obtained by their additive contribution at each time *t*:

$$\begin{array}{c} \dot{D}\left(t\right)={\dot{D}}_{fail}\left(t\right)+{\dot{D}}_{reco}\left(t\right)=\frac{B}{1+e^{Bt}}-\frac{A}{1+e^{-At}}. \end{array}$$ (6)

The last differential equation can be quickly solved by noting that a dysfunction indicator defined by

$$\begin{array}{c} D(t)=-\text{log}\;[f(t)g(t)], \end{array}$$ (7)

which leads to

$$\begin{array}{c} \dot{D}\left(t\right)=-\frac{f^{\prime }\left(t\right)}{f(t)}-\frac{g^{\prime }\left(t\right)}{g(t)}, \end{array}$$ (8)

is the solution if *f*(*t*) = 1 + *e*^−*Bt*^ and *g*(*t*) = 1 + *e*^*At*^. It follows that we can write

$$\begin{array}{c} D\left(t\right)=-\text{log}\;[\left(1+e^{-Bt}\right)\left(1+e^{At}\right)] \end{array}$$ (9)

and, by introducing the **shock function** $S\left(t\right)=-Me^{D(t)}$, where *M* is a parameter allowing one to shift the dysfunction to account for more general deviations from the baseline, it follows

$$\begin{array}{c} S\left(t\right)=-\frac{M}{\left(1+e^{-Bt}\right)\left(1+e^{At}\right)}. \end{array}$$ (10)

It is worth mentioning that logistic growth, underlying our hypothesis, is strictly related to epidemic models such as the Susceptible-Infected-Susceptible (SIS) and can be obtained from the widely used Susceptible-Infected-Recovered (SIR) model under the large susceptible population approximation [53, 54].

**Fig 2:**
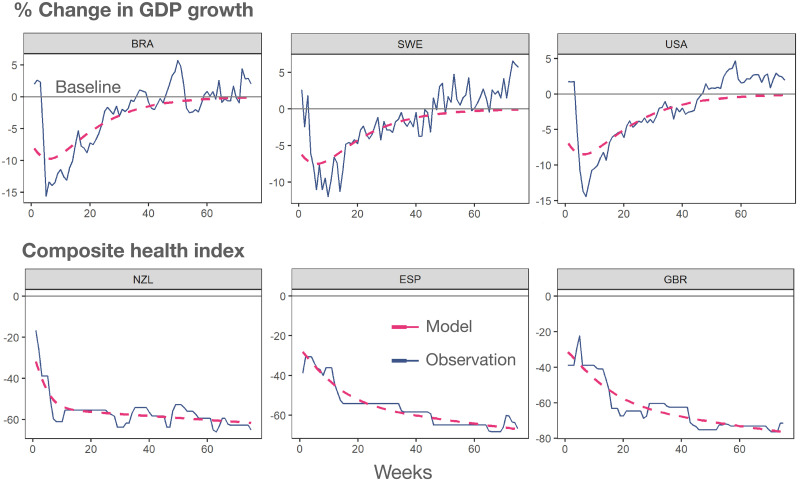
Agreement between model and country-level change indices. Observed time course of two distinct indices, economic (top) and health (bottom), for six distinct countries worldwide. The dashed line indicates the fit obtained through the corresponding shock function introduced in this work.

Fig 1 shows the shock function fitted to reproduce the behavior of the comprehensive shock index for a variety of countries, while Fig 2 shows the relation between parameters *A*, *B* and *M*. Table 1 reports the values of the parameters for all countries analyzed in this study.

**Table 1:** Model parameters. Numerical estimates of model parameters for each country, obtained by non-linear least squares analysis on the log transformation of the global shock index. BIC indicates the Bayesian Information Criterion indicator.

Country	M	B	A	BIC	Strategy
ARG	140.03	0.0099	0.0068	-296.85	Reactive
AUS	123.47	0.0095	0.0067	-326.54	Proactive
AUT	135.39	0.0094	0.0063	-238.84	Reactive
BEL	139.97	0.0088	0.0064	-277.35	Reactive
BGR	133.61	-0.0066	-0.0098	-199.95	Reactive
BRA	138.22	-0.0067	-0.0094	-269.45	Reactive
CAN	134.64	-0.0063	-0.009	-285.32	Reactive
CHE	134.86	0.0092	0.0063	-282.64	Reactive
CHL	140.29	-0.0067	-0.0097	-334.4	Reactive
COL	139.3	0.0099	0.007	-286.43	Reactive
CZE	136.1	-0.0061	-0.0094	-233.93	Reactive
DEU	134	0.0093	0.0063	-269.15	Reactive
DNK	129.67	0.0092	0.0063	-290.78	Reactive
ESP	140.23	0.0086	0.0064	-232.99	Reactive
EST	127.4	0.0096	0.0063	-212.74	Reactive
FIN	125.57	0.0093	0.0065	-286.16	Reactive
FRA	139.08	-0.0063	-0.0086	-221.81	Reactive
GBR	140.2	0.0091	0.0065	-293.18	Reactive
GRC	134.15	-0.0063	-0.0095	-186.44	Reactive
HUN	135.71	0.0096	0.0064	-215.38	Reactive
IDN	126.62	0.0102	0.0068	-250.72	Reactive
IND	130.58	0.01	0.0069	-260.91	Reactive
IRL	135.66	0.009	0.0064	-275.85	Reactive
ISR	134.02	0.0095	0.0065	-275.63	Reactive
ITA	141.77	-0.0062	-0.0084	-216.55	Reactive
JPN	120.39	0.0103	0.0066	-287.05	Reactive
KOR	119.04	0.01	0.0067	-346.1	Proactive
LTU	132.06	0.0098	0.0064	-221.29	Reactive
LUX	135.1	0.0093	0.0065	-247.36	Reactive
LVA	130.9	0.0094	0.0061	-261.43	Reactive
MEX	137.9	0.0098	0.0068	-323.22	Reactive
NLD	135.11	-0.0063	-0.0089	-313.11	Reactive
NOR	125.01	0.0092	0.0063	-270.93	Reactive
NZL	114.11	0.0094	0.0072	-289.59	Proactive
POL	133.92	-0.0064	-0.0095	-204.01	Reactive
PRT	137.98	-0.0063	-0.0091	-212.78	Reactive
ROU	134.93	0.0095	0.0064	-210.98	Reactive
RUS	131.45	0.0095	0.0064	-261.13	Reactive
SVK	135.04	-0.0063	-0.0098	-211.91	Reactive
SVN	136.51	0.0094	0.0063	-239.4	Reactive
SWE	135.17	-0.0063	-0.009	-342.45	Reactive
TUR	131.49	0.0094	0.0065	-221.61	Reactive
USA	137.63	-0.0064	-0.0092	-327.78	Reactive
ZAF	136.21	0.0096	0.0067	-266.25	Reactive

## Results

The interdependency between health, social and economic systems characterizes our society as a system of systems [55, 56] which can be studied under the lens of complexity science. Any system of interest has some (unknown) structure of interdependencies that it is exposed to shocks of various nature. It is plausible to assume that perturbations will propagate through the system, altering its function—measured by some systemic indicator *S*(*t*)—through a multiplicative cascade process [57], where a change at time *t* triggers even larger changes at a subsequent time *t* + Δ*t*, resulting in a quick decrease in the value of *S*(*t*). This first phase defines the failure of a system, and it can be modeled by a logistic-like expansion (see Materials and methods), as shown in Fig 1A, characterized by a carrying capacity accounting for the fact that resources that can be damaged are finite. For instance, in the case of the economic system, the systemic failure can be measured by relative changes in the GDP growth and employment levels of a country. (Choice of a specific systemic indicator such as GDP is left to the policy maker as it is inevitably laden with value judgments. The same can be said about the timescale of evaluation. Here, we have chosen GDP not because it is necessarily the best systemic indicator but because it is among the most widely used and referenced to in policy analysis). At some point, failures can no longer propagate either because there are no more resources or because mitigation procedures have been adopted: at this time step, *t*_*peak*_, the systemic indicator *S*(*t*) reaches a global minimum followed by an increase, which indicates that the system is bouncing back towards its fully operational state. This change of regime marks the beginning of a recovery phase and can be characterized by another logistic-like expansion, where one positive intervention on the system at time *t* facilitates subsequent interventions at the subsequent time *t* + Δ*t*. The overall process, detailed in Materials and Methods, leads to the model schematically reproduced in Fig 1A, which is described by the shock function

$$\begin{array}{c} S\left(t\right)=-\frac{M}{\left(1+e^{-Bt}\right)\left(1+e^{At}\right)}, \end{array}$$ (11)

where *M*, *A* and *B* are parameters to be estimated from empirical observations. The area under the two curves before and after the global minimum *S*_*peak*_ = *S*(*t*_*peak*_) can be used to quantify the robustness and the resilience of the underlying system to a given shock. If we define the total damage at time *t* as

$$\begin{array}{c} \Delta \left(t\right)=\int_{t_{0}}^{t}S\left(\tau \right)\;d\tau , \end{array}$$ (12)

the robustness of a system is related to the share of the area covered until the peak is reached, i.e.

$$\begin{array}{c} \epsilon \left(t\right)=1-\frac{\int_{t_{0}}^{t_{peak}}S\left(\tau \right)\;d\tau }{\Delta (t)}. \end{array}$$ (13)

If the system had a perfect response to the shock, the shock function is expected to be a Dirac delta function, suggesting an immediate recovery, the failure phase being characterized by an area under the curve equal to zero and the robustness index being equal to 1. Note that the case where the system remains substantially unaffected by the shock is compatible with the lack of a shock, by definition.

Conversely, if the system instantaneously collapses, the robustness index equals 0. Similarly, we can define the resilience of a system by the share of the area covered after the peak is reached, i.e.

$$\begin{array}{c} \gamma \left(t\right)=1-\frac{\int_{t_{peak}}^{t}S\left(\tau \right)\;d\tau }{\Delta (t)}. \end{array}$$ (14)

A system which instantaneously recovers to its original function will be characterized by a resilience index equal to 1. In practice, the two measures are not independent and one can expect the existence of a trade-off between robust response and fast recovery. Fig 1B shows how the relationship between robustness and resilience can be used to understand the response of different countries over a defined time window, or their evolution over time (Fig 1C).

We consider a broad set of indicators at country level, covering health, social, behavioral and economic aspects of systemic response to the COVID-19 pandemic. Specifically, we consider closure and health indices, built by accounting for several policies such as school closing, restrictions on gatherings, testing, contract tracing, etc. (see Materials and methods), for a total of 8 indicators for closure and 6 indicators for health as developed by the Oxford COVID-19 Government Response Tracker [49]. The behavioral index is obtained from Google human mobility anonymous and aggregate data [51]—already used to estimate optimal mobility reduction for the mitigation of COVID-19 transmission [58]—in terms of the median of the percent change from baseline value across six distinct types of movements: Retail and recreation, Grocery and pharmacy, Parks, Public transport stations, Workplaces and Residential. The economic index is built from the OECD Weekly Tracker [50], providing the percent change in weekly GDP levels from the pre-crisis trend. The epidemic index is obtained from the cumulative number of confirmed deaths due to COVID-19 relative to the total population in 2020 multiplied by one million [59].

**Fig 3:**
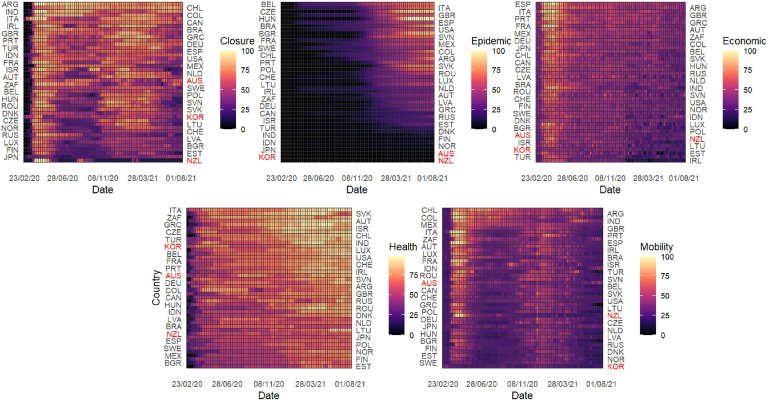
Indices considered to characterize the response of distinct countries to COVID-19. For all countries, the time course of the five indices is shown for the observational time between February 23, 2020 and August 01, 2021. Note that the country codes are reported on both sides of each heatmap to enhance the readability of the figure.

To allow for a comparison across countries, we have normalized the observational period between February 23, 2020 and August 1, 2021, where we have information for 44 countries worldwide. As an emblematic example, we show in Fig 2 the time course of the economic index and the composite health index for six countries, together with the corresponding shock functions, showing a nice agreement between data and expectation. Fig 3 shows the comparison of each index across all considered countries, for completeness.

**Fig 4:**
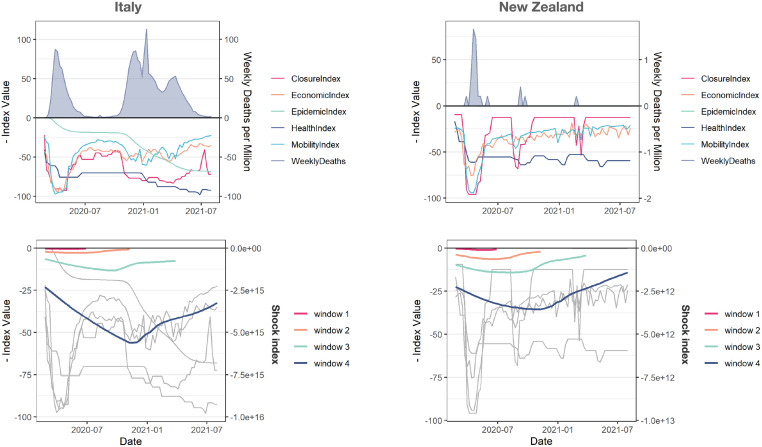
The shock index allows to compare the response of distinct countries to COVID-19. For two countries, Italy (left) and New Zealand (right), the time course of the six indices (top) and their convolution into a comprehensive shock index (bottom) is shown, for distinct values of the time window indicating a 19-weeks incremental length of the observational time between February 23, 2020 and August 01, 2021.

**Fig 5:**
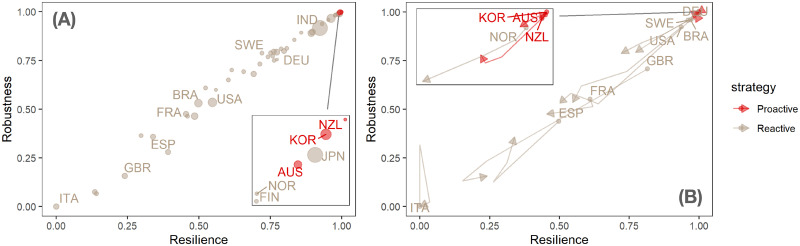
Proactive vs reactive strategies to COVID-19 pandemic. (A) static shock map where each point indicates a country and colors code proactive vs reactive response strategies. Remarkably, proactive strategies have a clear advantage, although some countries with reactive strategies, such as Nordic ones (except for Sweden) and Japan, perform similarly in robustness and resilience. (B) For a subset of countries, the evolution of their shock response over time is shown at different temporal snapshots.

However, while it is possible to compare robustness and resilience of countries with respect to a single indicator, it is still difficult to compare countries by means of a comprehensive index, to capture the multifaceted aspects of country-level responses. To overcome this issue, for each country separately we perform a convolution of the time course of its indices to obtain an overall shock index which harmonizes the heterogeneous signals that we consider. The result of this mathematical operation is shown in Fig 4 for two representative countries, namely Italy and New Zealand. The absolute value of the shock index in the two cases, obtained from the convolution of the aforementioned indices, reveals three orders of magnitude of difference in the overall response, with New Zealand exhibiting both higher robustness and higher resilience than Italy. We extend this analysis to all countries in our data set and show the results in Fig 5.

Fig 5 shows that the large majority of countries in our sample has opted for a reactive rather than a proactive strategy. However, the few countries adopting a proactive strategy consistently outperform the others both in terms of robustness and resilience. A few reactive countries feature levels of performance that are comparable to those of proactive ones. However, proactive countries better preserve their performance over time whereas similarly performing reactive ones slide down. Moreover, performances across reactive countries are very different, as shown for instance by EU countries, all of which took a reactive approach, but with very different results (Nordic countries generally do better than Mediterranean ones). The performances of proactive countries are instead very similar. Notice that geographical factors such as insularity do not have a clear effect on performance, as shown by the comparative results of the proactive New Zealand vs. the reactive UK. Clearly, the implementation of a proactive strategy in a non-insular geographical context may call for particularly severe measures, such as temporary mobility bans from and toward certain countries, and/or mandatory quarantines. Such measures might be unpopular and this might in turn influence the propensity of non-insular countries to adopt proactive rather than reactive strategies. Likewise, performance is not critically affected by levels of socio-economic development: for instance, US and Brazil fare quite similarly, and India largely outperforms both. The fact that our model, leading to the shock function (see Materials and methods for details), perfectly reproduces the behavior of the comprehensive shock index, provides interesting insights about the mechanisms—based on multiplicative growth processes—behind systemic failure and recovery of a country in response to external shocks such as the COVID-19 pandemic.

It is worth wondering about the results obtained from a similar analysis when one considers each index separately, rather than the composite index as we do. We report the result of this analysis in Fig 4, which highlights the lack of a well defined cluster of proactive countries and also suggests that much more information is encoded in the composite index than single indices in isolation.

## Discussion

Our results seem to deliver some clear messages that may be relevant for future policy design in response to pandemic shocks. First of all, proactive strategies seem strongly preferable to reactive ones as their immediate and anticipatory response curbs the diffusion of the virus and prevents the amplification of major socio-economic effects, as shown by ongoing research in the case of New Zealand [60]. Nevertheless, again for New Zealand, it has been also shown that a mitigation strategy might be more effective than elimination, if implemented in a mutated and more favorable context [61]. Very recently, it has been quantitatively shown for France that timely short strict draconian measures, such as lockdowns, are more effective and easier to control, as adherence wanes, than longer and moderate interventions [62], in agreement with our results on proactive strategies. This does not imply however that proactive strategies are in principle always preferable to reactive ones, and does not rule out the possibility that a well implemented reactive strategy could deliver equally good results in some circumstances. For instance, it has been recently shown that target isolation policies, where infectious individuals are rewarded to isolate themselves, can be used to couple economic and epidemic models for more effective control strategies [63]. Beyond direct economic effects, a longitudinal analysis of data from 15 countries has recently shown that elimination strategies are more effective in reducing transmission and deaths, while minimizing potential mental health effects [64] which play an important role for individuals and society [65].

Second, although in principle there should be a trade-off between robustness and resilience, our data show that in practice they are almost perfectly correlated, allowing an almost strict ranking of the performance of countries. Moreover, the performance of countries cannot be accounted for by traditional metrics such as levels of socio-economic development, and seems to depend on still poorly understood structural factors. In particular, the fact that only a few countries were able to adopt superior proactive response strategies need not depend only on political choices. Implementing a proactive strategy calls for high levels of social governance that might not be attainable in all countries without targeted adjustments. The future unfolding of the COVID-19 pandemic is unknown, especially in the light of new emerging variants of concern such as Omicron, and it is more likely that the post-pandemic world will have to coexist with the virus, requiring a rapid fast massive vaccine roll-out programmes at a global scale, rather than national one, across most age groups [66].

However, in view of our results, it could be advisable that countries capitalize upon the policy lessons of the current pandemic, and focus upon setting the conditions for a timely adoption of proactive responses against likely future pandemic shocks.

## Conclusion

The main message of our paper is that both proactive and reactive strategies of pandemic response may have a rationale, and that there is not in principle a response strategy that is objectively superior to the other. However, the two strategies imply different organizational challenges, so that the choice of one over the other also depends on the characteristics of the specific socio-political and cultural environment of a given country. In our study we find countries that adopted a reactive strategy with good results, but this option calls for very high levels of institutional coordination that allow a prompt and targeted response to critical situations. Likewise, we find countries that successfully adopted proactive strategies. In this case, the main challenge is building a significant predictive capacity, which calls for extensive data analysis and constant anticipatory monitoring of the phenomenon (e.g. in terms of appearance of emerging viral variants [67]).

To better understand which kind of response strategies are likely to be more effective in a given national or regional context, it will therefore be important to integrate data analysis with socio-political and cultural analyses of local institutional systems, and in particular of their critical weaknesses and of their dimensions of excellence and untapped potentials. A complexity-informed, evidence-based policy evaluation therefore calls for a major joint effort of integration of a number of different sources of scientific and policy expertise. Pursuing this goal will allow not only to improve policy responses to large-scale, potentially disruptive events in locally specific ways, but also to formulate new research agendas where it will be possible to pose new questions and investigate new issues as singled out by the collective intelligence of many different specialists and by the consilient leveraging of knowledge from diverse, and often so far poorly communicating, disciplinary spheres.

## Supporting information

S1 Fig Observed time course of the global shock index for different countries. Dashed lines represent the corresponding shock function fit obtained by non-linear least squares fitting method. On the y-axis the natural logarithm of the shock index is used.
(EPS)

S2 Fig Model parameters. Estimates of model parameters for each country, obtained by non-linear least squares analysis on the log transformation of the global shock index. Gray dashed lines indicate values equal to zero. Left: scatter plot of *A* vs *B*. Right: scatter plot of *A* vs *M*, with different shapes for the sign of the parameter *B*. Colours distinguish *Proactive* from *Reactive* strategies.
(EPS)

S3 Fig Single-index estimation. Estimates of robustness and resilience for each country, obtained by calculating shock functions on single indices (Closure, Economic, Health, Mobility and epidemic) separately, rather than the composite index. Colours distinguish *Proactive* from *Reactive* strategies.
(EPS)

## References

[1] La Porte TR. Organized social complexity: challenge to politics and policy. Princeton University Press; 2015.

[2] Jervis R. System effects: Complexity in political and social life. Princeton University Press; 1998.

[3] Pentland A. Social Physics: How social networks can make us smarter. Penguin; 2015.

[4] Yang Z, Neal Pd, Abdollahian M. When feedback loops collide: a complex adaptive systems approach to modeling human and nature dynamics. In: Advances in Applied Digital Human Modeling and Simulation. Springer; 2017. p. 317–327.

[5] Tran M. A general framework for analyzing techno-behavioural dynamics on networks. Environmental Modelling & Software. 2016;78:225–233. doi: 10.1016/j.envsoft.2015.12.004

[6] Wahlbeck K, McDaid D. Actions to alleviate the mental health impact of the economic crisis. World psychiatry. 2012;11(3):139. doi: 10.1002/j.2051-5545.2012.tb00114.x 23024664 PMC3449359

[7] Macintyre A, Ferris D, Gonçalves B, Quinn N. What has economics got to do with it? The impact of socioeconomic factors on mental health and the case for collective action. Palgrave Communications. 2018;4(1):1–5. doi: 10.1057/s41599-018-0063-2

[8] Qiu W, Rutherford S, Mao A, Chu C. The pandemic and its impacts. Health, culture and society. 2017;9:1–11. doi: 10.5195/HCS.2017.221

[9] Huremović D. Brief history of pandemics (pandemics throughout history). In: Psychiatry of pandemics. Springer; 2019. p. 7–35.

[10] Wölfel R, Corman VM, Guggemos W, Seilmaier M, Zange S, Müller MA, et al. Virological assessment of hospitalized patients with COVID-2019. Nature. 2020;581(7809):465–469. doi: 10.1038/s41586-020-2196-x 32235945

[11] Sun K, Wang W, Gao L, Wang Y, Luo K, Ren L, et al. Transmission heterogeneities, kinetics, and controllability of SARS-CoV-2. Science. 2021;371 (6526). doi: 10.1126/science.abe2424 33234698 PMC7857413

[12] Kraemer MUG, Hill V, Ruis C, Dellicour S, Bajaj S, McCrone JT, et al. Spatiotemporal invasion dynamics of SARS-CoV-2 lineage B.1.1.7 emergence. Science. 2021;373(6557):889–895. doi: 10.1126/science.abj0113 34301854 PMC9269003

[13] Davies NG, Abbott S, Barnard RC, Jarvis CI, Kucharski AJ, Munday JD, et al. Estimated transmissibility and impact of SARS-CoV-2 lineage B. 1.1. 7 in England. Science. 2021;372 (6538). doi: 10.1126/science.abg3055 33658326 PMC8128288

[14] Zhang J, Litvinova M, Wang W, Wang Y, Deng X, Chen X, et al. Evolving epidemiology and transmission dynamics of coronavirus disease 2019 outside Hubei province, China: a descriptive and modelling study. The Lancet Infectious Diseases. 2020;20(7):793–802. doi: 10.1016/S1473-3099(20)30230-9 32247326 PMC7269887

[15] Davis JT, Chinazzi M, Perra N, Mu K, y Piontti AP, Ajelli M, et al. Cryptic transmission of SARS-CoV-2 and the first COVID-19 wave. Nature. 2021. doi: 10.1038/s41586-021-04130-w PMC8636257 34695837

[16] Pullano G, Di Domenico L, Sabbatini CE, Valdano E, Turbelin C, Debin M, et al. Underdetection of cases of COVID-19 in France threatens epidemic control. Nature. 2021;590(7844):134–139. doi: 10.1038/s41586-020-03095-6 33348340

[17] Rockx B, Kuiken T, Herfst S, Bestebroer T, Lamers MM, Munnink BBO, et al. Comparative pathogenesis of COVID-19, MERS, and SARS in a nonhuman primate model. Science. 2020;368(6494):1012–1015. doi: 10.1126/science.abb7314 32303590 PMC7164679

[18] Ghavasieh A, Bontorin S, Artime O, Verstraete N, De Domenico M. Multiscale statistical physics of the pan-viral interactome unravels the systemic nature of SARS-CoV-2 infections. Communications Physics. 2021;4(1):1–13. doi: 10.1038/s42005-021-00582-8

[19] Gordon DE, Hiatt J, Bouhaddou M, Rezelj VV, Ulferts S, Braberg H, et al. Comparative host-coronavirus protein interaction networks reveal pan-viral disease mechanisms. Science. 2020;370 (6521). doi: 10.1126/science.abe9403 33060197 PMC7808408

[20] Zhang J, Litvinova M, Liang Y, Wang Y, Wang W, Zhao S, et al. Changes in contact patterns shape the dynamics of the COVID-19 outbreak in China. Science. 2020;368(6498):1481–1486. doi: 10.1126/science.abb8001 32350060 PMC7199529

[21] Perra N. Non-pharmaceutical interventions during the COVID-19 pandemic: A review. Physics Reports. 2021;913:1–52. doi: 10.1016/j.physrep.2021.02.001 33612922 PMC7881715

[22] Chinazzi M, Davis JT, Ajelli M, Gioannini C, Litvinova M, Merler S, et al. The effect of travel restrictions on the spread of the 2019 novel coronavirus (COVID-19) outbreak. Science. 2020;368(6489):395–400. doi: 10.1126/science.aba9757 32144116 PMC7164386

[23] Kraemer MU, Yang CH, Gutierrez B, Wu CH, Klein B, Pigott DM, et al. The effect of human mobility and control measures on the COVID-19 epidemic in China. Science. 2020;368(6490):493–497. doi: 10.1126/science.abb4218 32213647 PMC7146642

[24] Yang B, Huang AT, Garcia-Carreras B, Hart WE, Staid A, Hitchings MD, et al. Effect of specific non-pharmaceutical intervention policies on SARS-CoV-2 transmission in the counties of the United States. Nature communications. 2021;12(1):1–10. doi: 10.1038/s41467-021-23865-8 34117244 PMC8195990

[25] Lessler J, Grabowski MK, Grantz KH, Badillo-Goicoechea E, Metcalf CJE, Lupton-Smith C, et al. Household COVID-19 risk and in-person schooling. Science. 2021;372(6546):1092–1097. doi: 10.1126/science.abh2939 33927057 PMC8168618

[26] Maier BF, Brockmann D. Effective containment explains subexponential growth in recent confirmed COVID-19 cases in China. Science. 2020;368(6492):742–746. doi: 10.1126/science.abb4557 32269067 PMC7164388

[27] Dehning J, Zierenberg J, Spitzner FP, Wibral M, Neto JP, Wilczek M, et al. Inferring change points in the spread of COVID-19 reveals the effectiveness of interventions. Science. 2020;369 (6500). doi: 10.1126/science.abb9789 32414780 PMC7239331

[28] Kompas T, Grafton RQ, Che TN, Chu L, Camac J. Health and economic costs of early and delayed suppression and the unmitigated spread of COVID-19: The case of Australia. PloS one. 2021;16(6):e0252400. doi: 10.1371/journal.pone.0252400 34086731 PMC8177447

[29] König M, Winkler A. The impact of government responses to the COVID-19 pandemic on GDP growth: Does strategy matter? PloS one. 2021;16(11):e0259362. doi: 10.1371/journal.pone.0259362 34739509 PMC8570518

[30] Hayek S, Shaham G, Ben-Shlomo Y, Kepten E, Dagan N, Nevo D, et al. Indirect protection of children from SARS-CoV-2 infection through parental vaccination. Science. 2022;375(6585):1155–1159. doi: 10.1126/science.abm3087 35084938 PMC9799368

[31] Kofman A, Kantor R, Adashi EY. Potential COVID-19 endgame scenarios: eradication, elimination, cohabitation, or conflagration? Jama. 2021;326(4):303–304. doi: 10.1001/jama.2021.11042 34236382

[32] Oliu-Barton M, Pradelski BS, Algan Y, Baker MG, Binagwaho A, Dore GJ, et al. Elimination versus mitigation of SARS-CoV-2 in the presence of effective vaccines. The Lancet Global Health. 2022;10(1):e142–e147. doi: 10.1016/S2214-109X(21)00494-0 34739862 PMC8563003

[33] Dowdle WR. The principles of disease elimination and eradication. Bulletin of the World Health Organization. 1998;76(Suppl 2):22. 10063669 PMC2305684

[34] Vindegaard N, Benros ME. COVID-19 pandemic and mental health consequences: Systematic review of the current evidence. Brain, behavior, and immunity. 2020;89:531–542. doi: 10.1016/j.bbi.2020.05.048 32485289 PMC7260522

[35] Farboodi M, Jarosch G, Shimer R. Internal and external effects of social distancing in a pandemic. Journal of Economic Theory. 2021;196:105293. doi: 10.1016/j.jet.2021.105293

[36] Walmsley TL, Rose A, Wei D. Impacts on the US macroeconomy of mandatory business closures in response to the COVID-19 Pandemic. Applied Economics Letters. 2021;28(15):1293–1300. doi: 10.1080/13504851.2020.1809626

[37] Perry BL, Aronson B, Pescosolido BA. Pandemic precarity: COVID-19 is exposing and exacerbating inequalities in the American heartland. Proceedings of the National Academy of Sciences. 2021;118(8). doi: 10.1073/pnas.2020685118 33547252 PMC7923675

[38] Jord‘a O, Singh SR, Taylor AM. Longer-run economic consequences of pandemics? The Review of Economics and Statistics. 2020; p. 1–29.

[39] Haderlein SK, Saavedra AR, Polikoff MS, Silver D, Rapaport A, Garland M. Disparities in Educational Access in the Time of COVID: Evidence From a Nationally Representative Panel of American Families. Aera Open. 2021;7:23328584211041350. doi: 10.1177/23328584211041350

[40] Nicola M, Alsafi Z, Sohrabi C, Kerwan A, Al-Jabir A, Iosifidis C, et al. The socio-economic implications of the coronavirus pandemic (COVID-19): A review. International journal of surgery. 2020;78:185–193. doi: 10.1016/j.ijsu.2020.04.018 32305533 PMC7162753

[41] Barlow J, Vodenska I. Socio-Economic Impact of the COVID-19 Pandemic in the US. Entropy. 2021;23(6):673. doi: 10.3390/e23060673 34071928 PMC8227473

[42] Delardas O, Kechagias KS, Pontikos PN, Giannos P. Socio-Economic Impacts and Challenges of the Coronavirus Pandemic (COVID-19): An Updated Review. Sustainability. 2022;14(15):9699. doi: 10.3390/su14159699

[43] Iacus SM, Natale F, Santamaria C, Spyratos S, Vespe M. Estimating and projecting air passenger traffic during the COVID-19 coronavirus outbreak and its socio-economic impact. Safety Science. 2020;129:104791. doi: 10.1016/j.ssci.2020.104791 32377034 PMC7200368

[44] Collins-Kreiner N, Ram Y. National tourism strategies during the Covid-19 pandemic. Annals of tourism research. 2020;. doi: 10.1016/j.annals.2020.103076 33100431 PMC7572067

[45] Marino L, Capone V. Smart working and well-being before and during the COVID-19 pandemic: A scoping review. European Journal of Investigation in Health, Psychology and Education. 2021;11(4):1516–1536. doi: 10.3390/ejihpe11040108 34940386 PMC8700761

[46] Lenzen M, Li M, Malik A, Pomponi F, Sun YY, Wiedmann T, et al. Global socio-economic losses and environmental gains from the Coronavirus pandemic. PloS one. 2020;15(7):e0235654. doi: 10.1371/journal.pone.0235654 32645023 PMC7347123

[47] Manipis K, Street D, Cronin P, Viney R, Goodall S. Exploring the trade-off between economic and health outcomes during a pandemic: a discrete choice experiment of lockdown policies in Australia. The Patient-Patient-Centered Outcomes Research. 2021;14(3):359–371. doi: 10.1007/s40271-021-00503-5 33694076 PMC7946575

[48] Hausmann R, Schetter U. Horrible trade-offs in a pandemic: Poverty, fiscal space, policy, and welfare. World Development. 2022;153:105819. doi: 10.1016/j.worlddev.2022.105819

[49] Hale T, Angrist N, Goldszmidt R, Kira B, Petherick A, Phillips T, et al. A global panel database of pandemic policies (Oxford COVID-19 Government Response Tracker). Nature Human Behaviour. 2021;5(4):529–538. doi: 10.1038/s41562-021-01079-8 33686204

[50] Woloszko N. A Weekly Tracker of activity based on machine learning and Google Trends; 2021.

[51] Google. Human mobility changes at national level. Google Mobility Report. 2021;.

[52] Ritchie H, Roser M. Age Structure. Our World in Data. 2019;.

[53] Murray JD. Mathematical Biology I. An Introduction. vol. 17 of Interdisciplinary Applied Mathematics. 3rd ed. New York: Springer; 2002.

[54] Pastor-Satorras R, Castellano C, Van Mieghem P, Vespignani A. Epidemic processes in complex networks. Reviews of modern physics. 2015;87(3):925. doi: 10.1103/RevModPhys.87.925

[55] Buldyrev SV, Parshani R, Paul G, Stanley HE, Havlin S. Catastrophic cascade of failures in interdependent networks. Nature. 2010;464(7291):1025–1028. doi: 10.1038/nature08932 20393559

[56] Barabási AL. The network takeover. Nature Physics. 2012;8(1):14–16. doi: 10.1038/nphys2188

[57] Dorogovtsev SN, Goltsev AV, Mendes JF. Critical phenomena in complex networks. Reviews of Modern Physics. 2008;80(4):1275. doi: 10.1103/RevModPhys.80.1275

[58] Nouvellet P, Bhatia S, Cori A, Ainslie KE, Baguelin M, Bhatt S, et al. Reduction in mobility and COVID-19 transmission. Nature communications. 2021;12(1):1–9. doi: 10.1038/s41467-021-21358-2 33597546 PMC7889876

[59] Ritchie H, Mathieu E, Rodés-Guirao L, Appel C, Giattino C, Ortiz-Ospina E, et al. Coronavirus Pandemic (COVID-19). Our World in Data. 2021;.

[60] Wilson N, Grout L, Summers JA, Nghiem N, Baker MG. Use of the elimination strategy in response to the COVID-19 pandemic: health and economic impacts for New Zealand relative to other OECD countries. medRxiv. 2021;.

[61] Lally M. The costs and benefits of COVID-19 lockdowns in New Zealand. medRxiv. 2021;.

[62] Domenico LD, Sabbatini CE, Boëlle PY, Poletto C, Crépey P, Paireau J, et al. Adherence and sustainability of interventions informing optimal control against the COVID-19 pandemic. Communications Medicine. 2021;1(1). doi: 10.1038/s43856-021-00057-5 35602184 PMC9053235

[63] Ash T, Bento AM, Kaffine D, Rao A, Bento AI. Disease-economy trade-offs under alternative epidemic control strategies. Nature Communications. 2022;13(1):1–14. doi: 10.1038/s41467-022-30642-8 PMC9178341 35680843

[64] Aknin LB, Andretti B, Goldszmidt R, Helliwell JF, Petherick A, De Neve JE, et al. Policy stringency and mental health during the COVID-19 pandemic: a longitudinal analysis of data from 15 countries. The Lancet Public Health. 2022;7(5):e417–e426. doi: 10.1016/S2468-2667(22)00060-3 35461592 PMC9023007

[65] Layard R, Clark DM. Why more psychological therapy would cost nothing; 2015. doi: 10.3389/fpsyg.2015.01713 26635648 PMC4658447

[66] Marziano V, Guzzetta G, Mammone A, Riccardo F, Poletti P, Trentini F, et al. The effect of COVID-19 vaccination in Italy and perspectives for living with the virus. Nature Communications. 2021;12(1):1–8. doi: 10.1038/s41467-021-27532-w 34907206 PMC8671442

[67] Klamser P, d’Andrea V, Di Lauro F, Zachariae A, Bontorin S, di Nardo A, et al. Enhancing global preparedness during an ongoing pandemic from partial and noisy data. medRxiv. 2022. doi: 10.1101/2022.08.19.22278981 PMC10282504 37351112

